# Online Games and Cognitive Distortions: A Comparative Analysis in Students with and without Disabilities

**DOI:** 10.3390/ejihpe14070123

**Published:** 2024-06-24

**Authors:** Raquel Suriá-Martínez, Fernando García-Castillo, Carmen López-Sánchez, Esther Villegas, Carmen Carretón

**Affiliations:** 1 Department of Comunication and Social Psychology, Universidad de Alicante, 03690 San Vicente del Raspeig, Spain; mc.lopez@ua.es (C.L.-S.); mc.carreton@ua.es (C.C.); 2 Departament of Educatión, Universidad de Alicante, 03690 San Vicente del Raspeig, Spain; f.garciacastillo@ua.es; 3 Department of Social Work and Social Affairs, Universidad de Alicante, 03690 San Vicente del Raspeig, Spain; esther.villegas@ua.es

**Keywords:** online games, addiction, misbeliefs, students, disability, intervention

## Abstract

Online games have experienced significant growth in recent years, with gaming becoming a popular form of entertainment for people of all ages. However, their impact on cognition, especially among vulnerable groups such as students with disabilities, is a topic that requires deeper exploration. The objectives of this study are twofold: firstly, to understand the typology of risk players (non-risk players, players with problems, and pathological players); and secondly, to compare cognitive distortions among students with problematic profiles. Both objectives will be analyzed based on the presence or absence of disability. A total of 704 students from various Spanish universities (135 with disabilities and 569 without disabilities), aged between 18 and 38, participated in the study by completing the Gamblers Belief Questionnaire (GBQ), aimed at measuring cognitive distortions related to gambling problems, as well as the Massachusetts Gambling Screen questionnaire, aimed at measuring gambling addiction. The results indicate a higher percentage of students with disabilities showing a greater risk profile for addiction. Additionally, this group of students exhibits more cognitive distortions. These findings underscore the need for a comprehensive approach to addressing online gaming addiction and cognitive distortions among university students, with and without disabilities. Preventive measures are necessary, such as education on responsible technology use and the promotion of alternative activities. Moreover, specific intervention strategies need to be developed, including access to psychological health services for this student population.

## 1. Introduction

Gambling, from sports betting to online casinos, has long been a popular form of entertainment, with its popularity further developing with the expansion of online platforms and mobile applications [1,2,3,4]. However, along with its growth, concerns have arisen about the negative impact it may have on individuals’ mental health, especially on university students [2,5]. One of the greatest risks is the development of compulsive gambling problems, which can negatively affect mental health and academic performance. Students may fall into a cycle of excessive gambling, neglecting their academic and social responsibilities in favor of the thrill of the game [6,7].

In this regard, online gambling refers to any form of betting or games of chance conducted over the Internet. This encompasses a wide range of activities such as virtual casinos, sports betting, online poker, bingo, and lotteries. Players access these digital platforms using internet-connected devices like computers, smartphones, or tablets. Gambling becomes pathological when an individual loses control over their gambling behavior, leading to significant negative consequences in their personal, social, and professional life.

Regarding prevalence in Spain, online gambling has seen a notable increase in popularity in recent years. According to reports from the “General Directorate for the Regulation of Gambling” (DGOJ) in Spain, participation in online gambling activities has steadily grown. In 2022, it was estimated that around 7.2% of the adult population in Spain had participated in some form of online gambling in the past 12 months. However, this trend has also raised concerns about the potential risks associated with problem gambling and addiction.

Moreover, engaging in gambling at a young age can establish problematic behavioral patterns that persist into adulthood. Gambling addiction can have devastating long-term consequences, including relationship breakdowns, financial problems, and legal issues [8,9].

Among the factors contributing to gambling participation among students, published literature reveals that easy accessibility and availability to online gambling platforms and aggressive advertising targeting young people are two of the main drivers. Additionally, peer pressure and the pursuit of thrills may lead students to try their luck in gambling [5]. Academic stress and personal difficulties may also lead some students to seek escape via gambling. Lack of awareness of the risks associated with gambling and the mistaken belief that gambling is a quick way to make money may also influence students’ participation in these activities [2].

While the relationship between gambling and youth in general has been widely researched, the development of the Internet and the growing proliferation of online gambling may be a more attractive form of entertainment for certain groups of students, particularly those with disabilities. In this regard, although there is a lack of specific attention to how participation in these spaces may affect students living with disabilities, limited research suggests that students with disabilities may be more vulnerable to problematic gambling due to a variety of factors [10,11]. For example, these students may have difficulties participating in conventional social activities, which could lead them to seek entertainment in gambling, especially if it is readily available in their virtual environment. Additionally, the lack of opportunities for inclusive and social leisure activities, the search for emotions, and a lack of awareness of the associated risks may contribute to gambling among students with disabilities.

Regardless of disability, the world of gambling, whether in casinos, online, or otherwise, can be fertile ground for a variety of cognitive distortions [12,13,14]. These distortions are irrational thought patterns that can lead to misinterpretations of reality and affect decision-making. In the context of gamblers, these distortions can be especially problematic, as they can contribute to compulsive and addictive behaviors [15,16]. Thus, there are several cognitive variables that the literature indicates are associated with gambling participation and may influence the level of participation and the risk of its abusive use among students in general [10,17].

Among the most common cognitive distortions in this group are the so-called “gambler’s fallacy”, or the belief that past outcomes will influence future outcomes, for example, believing that after a series of losses, a win is guaranteed; “unrealistic optimism”, or the tendency to overestimate the chances of winning and underestimate the risks associated with gambling; the “illusion of control”, or the mistaken belief that one has control over the outcomes of the game when it is actually a purely random event; and, finally, “selective memory”, which is the tendency to remember wins and forget losses, leading to a distorted perception of one’s gambling history [18,19].

These cognitive distortions can be especially dangerous when combined with the addictive nature of some gambling games, twisting the perception of reality and negatively affecting the mental health and academic performance of disabled and non-disabled college students.

However, notwithstanding the importance of detecting these beliefs in students who participate in gambling, there is a gap in the research on the participation of students with disabilities in such activities which delves into preferences in the typology of games, as well as in determining whether the participation of these students proliferates more in an online format. In this sense, there are previous studies that highlight the disabled population as a vulnerable group to addictions, specifically problematic Internet use [10,11]. Additionally, the possible existence of differences in the participation profile between students with and without disabilities is unknown. Finally, it would be interesting to know the cognitive distortions of students and whether these differ depending on their disability or not.

Based on these considerations, the objectives of this study are twofold. First, the objective is to know the typology of at-risk players (non-risk players, at-risk players, and problem players) as a function of having or not having a disability. The second objective seeks to compare students’ cognitive distortions based on the typology of at-risk players. This will be examined on the basis of disability and non-disability.

From the first objective arises the first hypothesis:

**H.1.**  *Different risk profiles will be observed, with a higher incidence of problematic cases among students with disabilities.*

From the second objective, the following hypothesis is proposed:

**H.2.**  *Greater cognitive distortions will be found in the problematic profiles, with higher scores observed in the group of students with disabilities.*

## 2. Materials and Methods

### 2.1. Participants

This study is based on the analysis of data collected from a sample of 704 university students from Spanish universities. Participants were selected through convenience sampling and an open online questionnaire during the months of January to March 2024. Regarding the composition of the sample, 24.9% were female (175 participants) and 75.1% were male (529 participants). The age of the participants ranged from 18 to 33 years, distributed as follows: 24.1% were between 18 and 22 years old, 38.9% between 23 and 27 years old, and 37% between 28 and 33 years old. In terms of educational level, 93.5% were undergraduate students, with 31.1% in the first year, 14.3% in the second year, 12.6% in the third year, and 35.4% in the fourth year, while 6.5% were postgraduate and/or master’s degree students. Additionally, 18.5% of the sample (130 participants) reported having some form of recognized disability, distributed as follows: 8.7% motor, 3.7% mental, 3.0% auditory, 1.7% cognitive, and 1.4% visual.

### 2.2. Procedure

Data collection was conducted by administering a questionnaire to a group of selected students between January and March 2024. The sample was drawn from various academic programs across different Spanish universities. Before inviting students to participate, contact was made with academic authorities to have the questionnaire link published on the university’s webpage. Additionally, psychological support services were contacted to collaborate in disseminating the questionnaire to students with and without disabilities. The questionnaire was hosted on Google Forms and advertised on campus. The estimated time to complete the questionnaire was approximately 10 min.

Prior to data collection, the study underwent ethical review by the ethics committee of the University of Alicante to ensure compliance with research ethics standards. This process included obtaining informed consent from participants and ensuring they fully understood the study’s objectives, procedures, and any potential risks or benefits associated with their participation. Measures were implemented to protect the privacy and confidentiality of the collected data. Approval from the ethics committee confirms that the study was designed and executed in a manner that respects the rights and well-being of the participants. Additionally, a commitment was made that the data would only be used for research purposes, maintaining anonymity.

### 2.3. Instruments

To determine the risk of pathological gambling, criteria from the DSM-V, ICD, and the SOG-RA scale [20] were followed, indicating risk ranges based on gambling activity (once or several times a year = non-risk player, several times a month or several times a week = at-risk player, once a day and several times a day = problem player).

To measure cognitive distortions related to gambling problems (Table 1), an adaptation of the Gamblers Belief Questionnaire (GBQ) [21] was used. In the present research, unlike the original questionnaire, it was reduced to 20 items measured on a scale of 1 = not at all to 5 = very/extreme. The internal consistency of the original GBQ version of the scale was 0.92, and the test-retest reliability was 0.77, obtaining two related factors: luck/perseverance luck/perseverance (α = 0.90) including items: 4, 6, 10, 11, 12, 13, 14, 15, 16, 17, 19 and 20; and illusion of control (α = 0.84) with items: 1, 2, 3, 5, 7, 8, 9 and 18.

The descriptive analysis of the 20 items included in the scale showed that six items had higher averages (tending towards value 3 = moderate) than the theoretical average (value 2 = a little): 1. “My choices affect the game I am betting on”; 7. “Gambling is more than just luck”; 8. “My wins in gambling are proof that I have skill and knowledge about the game”; 9. “I have a “lucky technique” that I use when I gamble”; 10. “In the long run, I will make more money than I can lose gambling”; and 17. “If I keep playing over time, I will be rewarded and make money”. The items with the highest and lowest scores were as follows: the highest for item 8. “My wins in gambling are proof that I have skill and knowledge about the game” from the illusion of control dimension; and the lowest for item 20. “I must keep the same bet even when it hasn’t come out lately because it is destined or linked to win” from the luck/perseverance dimension. The analysis of kurtosis coefficients revealed that no item showed a value greater than ±2 (considered unacceptable), 19 items were leptokurtic (data values were more concentrated around the mean) and there were fewer outliers (except for item 15. “It doesn’t matter where I get the money to gamble because I will win and return it”). The reliability analysis of the measurement scales of the 20 items grouped through Cronbach’s alpha (α = 0.982) was excellent.

An exploratory factor analysis using principal components and the Varimax orthogonal rotation method was first performed to demonstrate the internal validity of the scale structure of the items, obtaining plausible and feasible sample adequacy indices (KMO = 0.927; Bartlett, X^2^ = 25635.545, *p* ≤ 0.001). The factorial solution yielded two factors that jointly explained 83.7% of the variance of the data, the first of which explained 76.7% and the second, of a more residual nature, explained 7.1%. Subsequently, and to confirm the bidimensionality, the analysis was performed again using the maximum likelihood extraction method and again by Varimax rotation. All estimated parameters were statistically significant (*p* < 0.01), all the factor loadings were greater than 0.60, the goodness of fit of the model was evaluated through X^2^ being significant (*p* < 0.01), and to measure the reliability of the scale of each of the factors the Cronbach’s alpha coefficient was used, with the obtained indices being: α = 0.97 for Factor 1 and α = 0.76 for Factor 2.

### 2.4. Design

When selecting university students divided into two groups based on whether they have a disability or not, a cross-sectional design with an intentional and non-probabilistic approach was employed. This design is useful for researching specific groups in particular contexts, such as the university setting.

### 2.5. Data Analysis

To obtain the sociodemographic data, frequencies and percentages were calculated. The homogeneity test (X^2^) was used to compare the type of gambler at risk (non-risk, at-risk, and problem or pathological), according to the disability variable. To test whether cognitive distortions can predict gambling problems, a multinomial logistic regression model was applied. The dependent variable was the type of player according to the risk involved, grouped into three categories: non-risk, at-risk, and problem or pathological. The twenty items related to cognitive distortions grouped in the two factors resulting from the previous exploratory factor analysis were included as independent variables, and the disability condition variable (0 = person without disability; 1 = person with disability) was included as a factor of analysis. The stepwise backward method was used in the interaction, and players without risk were taken as the reference category. To compare the models obtained with the dependent variable, the AIC (Akaike) and SBC (Schwarz) selection criteria were employed. The final model (AIC = 899) was better adjusted (deviance = 0.149) than having only the B values, which were lower when compared. Regarding goodness of fit, the McFadden Pseudo-R^2^, used to see the size of the effect of the predictor variables on the dependent variable, yielded a value of 0.167, and although this is a very discrete value, the Cox and Snell test obtained a value of 0.261 and the Nagelkerke test of 0.312, so the model had a good fit and explained 31.2% of the change in the dependent variable. Likelihood ratio tests indicated the importance of the predictors in the model, and in this sense, students’ gender, disability status, and 10 out of the 20 included items of cognitive distortions were significant (sig. < 0.05).

## 3. Results

**Figure 1:**
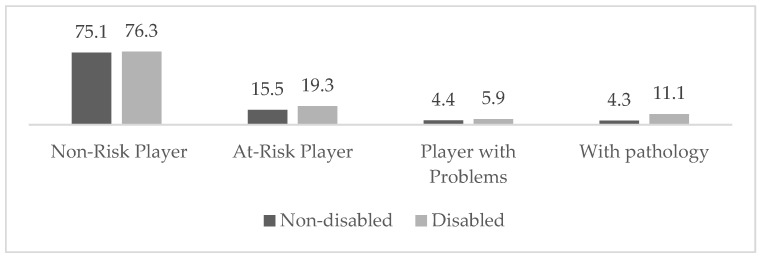
Homogeneity test (X^2^) of type of at-risk player based on disability status.

Regarding the first objective, the identification of the type of risky player showed that a high percentage is at risk of developing gambling problems (Figure 1), with approximately 4.7% already having problems, and 5.6% being addicted. Thus, regarding the type of risky player based on whether they have a disability or not, the analysis indicated the existence of statistically significant differences, although in all analyses the effect was medium (Phi ≤ 0.20): a higher percentage was observed in students with disabilities in problematic profiles. Thus, a profile of pathological players (11.1%) and at-risk players (5.9%) was observed (X^2^ = 14.035, *p* < 0.05, Phi = 1.141).

Regarding cognitive distortions based on the at-risk player typology, the analyses indicated the following results:

Comparative analysis: non-risk player vs. at-risk player. The condition of having a disability and four predictors of the cognitive distortions scale: “When I lose while gambling, it’s not so bad if I don’t tell people I care about” (F2), “I have more knowledge and skills related to gambling than most people who gamble” (F2), “Those who don’t gamble much don’t understand that success in gambling requires dedication and a willingness to invest some money” (F1), and “I should keep a record of the bets I’ve won before so I can figure out how I should bet in the future” (F1) significantly predicted (sig. < 0.05) the probability of a student transitioning from being a non-risk player to an at-risk one. Having a disability indicated a 2.5-times greater chance of transitioning to being an at-risk player (Exp B = 0.394) compared to not having a disability. Regarding the two main predictors of the cognitive distortions scale: “Those who don’t gamble much don’t understand that success in gambling requires dedication and a willingness to invest some money” (Exp B = 2.872), that is, as the value of the predictor increases by one unit, the student is 2.8-times more likely to transition to being an at-risk player, and in “I have more knowledge and skills related to gambling than most people who gamble,” there exists a 2.4-times greater chance for this type of player to transition.

Comparative analysis: non-risk player vs. problem player (Table 2). The condition of having a disability and four predictors of the cognitive distortions scale: “My choices affect the game I’m betting on” (F1), “I should keep a record of the bets I’ve won before so I can figure out how I should bet in the future” (F1), “When I’m playing and I’m about to lose or when I almost win, it reminds me that if I keep playing, I’ll win” (F1), and “Those who don’t gamble much don’t understand that success in gambling requires dedication and a willingness to invest some money” (F1) significantly predicted (sig. < 0.05) the probability of a student transitioning from being a non-risk player to one with problems. Having a disability indicates a 4.7-times greater chance to transition to a problem player (Exp B = 0.214) compared to not having a disability. Regarding the two main predictors of the scale, both associated with Factor 1 (luck/perseverance): “Those who don’t gamble much don’t understand that success in gambling requires dedication and a willingness to invest some money” (Exp B = 2.707), that is, as the value of the predictor increases by one unit, the student is 2.7-times more likely to transition to a problem player, and in “When I’m playing and I’m about to lose or when I almost win, it reminds me that if I keep playing, I’ll win” there exists a 0.3-times greater chance for this type of player to transition. In the disability-item interaction, changes were also predicted: among people with disabilities, a higher probability of transitioning to a problem player was observed compared to those without disabilities in the items “My knowledge and skills in the game contribute to the possibility of making money” (Exp B = 0.099) and “Those who don’t gamble much don’t understand that success in gambling requires dedication and a willingness to invest some money” (Exp B = 0.181), both associated with Factor 1 (luck/perseverance).

## 4. Discussion

This study focuses on researching participation in online gaming and gambling among university students, with special attention paid to how disability may affect this activity. Despite the fact that online gaming has been popular among college students for years, trends in this area are constantly changing.

Investigating gambling addiction in students with disabilities is crucial because there is virtually no published literature on gambling addiction in this group. However, this population may be more vulnerable to developing such addictions. The lack of access to traditional recreational activities, the need for social interaction, and the search for excitement can lead to an increased risk of gambling addiction in this group. Understanding the factors contributing to this addiction in students with disabilities can help develop specific prevention and treatment strategies tailored to this population.

Overall, the results reveal that, although there is not a high prevalence of problem gambling among the participants, there is a small group that dedicates several days a week to this activity. The increase in online gambling among this population is notable and may be influenced by various factors such as easy access to internet-connected devices and the wide availability of online games [22,23,24,25,26]. This increase is particularly noticeable among students with disabilities. In this regard, a Canadian study conducted by the authors of [27], which addressed gambling in students with intellectual and developmental disabilities, compared the risk of online gambling addiction between players with disabilities and non-players: a higher percentage of teenagers who were probable pathological gamblers reported having a learning disability (22.3% vs. 9.4%) or cognitive problems (42.5% vs. 13.6%).

As some authors highlight, online gaming and gambling platforms can offer accessibility advantages, making it easier for young people with disabilities to participate more frequently [28,29]. Furthermore, there are very common characteristics among people with disabilities that may make them more sensitive to the regular use of virtual spaces. For example, there is published literature indicating that young people with disabilities have a reduced social circle and feelings of loneliness [30,31,32]. This isolation may lead these young people to spend more time at home and, therefore, be more exposed to these online leisure activities [3,33].

At the same time, abusive participation in online gambling among students with disabilities may vary compared to those without disabilities due to other factors such as social opportunities, lack of alternative activities and the pursuit of excitement [3,34]. In this regard, several authors note that online games have emerged among young people as a form of entertainment and socialization, providing students with disabilities a way to connect with friends and engage in recreational activities without the physical limitations they may face in other social environments [11,29]. Likewise, online platforms can level the playing field for people with disabilities, providing opportunities for participation in such recreational activities that may be more difficult in physical environments. Finally, online gambling can serve as a way to escape the reality of their disability and the limitations they face in everyday life, providing a temporary distraction from the physical or emotional challenges encountered by students with this disability. All of this can lead to an ideal leisure context that can be accessed for leisure activities that do not involve added effort, as can occur with the leisure spaces available on a multitude of Internet sites [1,3,4].

Finally, another hypothesis has focused on the risk perception of students with disabilities, suggesting that this group may have a higher risk of developing gambling problems because the perceived risk of gambling addiction is lower in people with disabilities than in those without disabilities [35]. Along these lines, research found that people with disabilities, specifically those with cognitive disabilities, had a lower risk perception and a greater willingness to take risks compared to people without disabilities [29].

With the intention of deepening this relationship, different research suggests that people with disabilities may have a different perception of risk based on the hypothesis of the influence of factors such as self-concept, self-efficacy and the ability to fully understand the consequences of their actions [36]. Therefore, although it is true that the self-concept and self-efficacy of a person living with a disability develop similarly to those without this handicap, their development may be hampered if they receive negative references from their environment, having to face various situations of social rejection and negative experiences in their social and interpersonal relationships from childhood, which results in personal devaluation and their actions having no relevance in their future [37,38,39].

These results support those obtained by a study conducted by the authors of [40], which compared the risk of gambling addiction between players without disabilities and the population with this handicap. The results indicated a higher risk of developing gambling problems among participants with disabilities. Other research addressing gambling in people with disabilities comes from an unpublished presentation at the 2014 Annual Meeting of the American Psychiatric Association by Kalinowski (2014). The authors surveyed 79 patients attending a psychiatric clinic for adults with intellectual disabilities. In total, 67% had mild disability and 21% had moderate disability. Using adapted versions of the Gambling Symptom Assessment Scale and the Structured Clinical Interview for Pathological Gambling, 2.5% met the DSM-IV-TR criteria for pathological gambling, and others (6.3%) for problem gambling. Additionally, 88% of the sample had gambled in the past, 71% in the previous year, and 20% reported gambling weekly.

Finally, the authors of [27] compared the risk of gambling addiction between players with disabilities and non-players and found that a higher percentage of teenagers who were probable pathological gamblers reported having a learning disability (22.3% vs. 9.4%) or cognitive problems (42.5% vs. 13.6%). These authors suggested that these findings were justified by the different risk perceptions of gambling, as well as by a series of factors, such as stress, social isolation, a lack of alternative activities and barriers to accessing support services.

Regarding the analysis of cognitive distortions in students based on whether they have a disability and the risk of addiction, the results reflect differences between students with and without disabilities in some erroneous beliefs about gambling (e.g., the belief that “keeping records of their winnings will lead to future wins”, thinking that “those who do not gamble do not understand that success requires dedication and willingness to invest money” or the assumption that “they have more knowledge and skills than most people who gamble”). This is observed in both non-risk and problem gamblers with disabilities.

In this sense, it is important to recognize that cognitive distortions can affect any player, regardless of whether they have a disability. However, in the case of players with disabilities, these distortions may be more pronounced due to the additional barriers they face, such as discrimination, lack of accessibility in games and the lack of representation in the industry.

Connecting to the Internet can distort reality for people with disabilities for several reasons. For instance, the Internet can become a substitute for direct social interaction. This can lead to a distorted perception of relationships and social skills, as online communication lacks non-verbal cues and empathy found in face-to-face interactions. Additionally, individuals with disabilities may be exposed to misleading or biased information online, which can affect their perception of reality. This includes fake news, conspiracy theories, and misinformation that may be harder to discern without proper media literacy. This can distort their cognitive component, information, beliefs, and values, leading them to believe what they see on the Internet is true or accurate.

Moreover, the use of online games and virtual reality applications may lead some people with disabilities to prefer these environments over real life. Immersing oneself in these virtual worlds can result in a disconnection from physical reality and tangible human relationships. Lastly, easy access to the Internet can contribute to technological dependence and social isolation. This demographic may choose to stay at home and use the Internet instead of going out and participating in social activities, which can distort their expectations and perception of reality.

The results are in line with those obtained by the authors of [41], in which a large sample of Canadian students in special education (N = 266) were compared to those who were not (N = 1738) on measures of problematic gambling symptomatology and ADHD. Students in special education showed more erroneous beliefs about gambling than their peers with typical development and were also at greater risk for developing problematic gambling patterns. When analyzing the two factors of the scale, it is observed that students with disabilities reflect greater distortions in both risk and non-risk situations in both factors. Regarding Factor 1 (luck/perseverance), the analyses show statistically significant differences when comparing the scores of disabled players with non-disabled players. This is especially reflected in problem players. In this sense and given that they are players who participate daily and/or several times a week, it is logical that they have had successful experiences in the game despite their disability. This, along with the beliefs of the Factor 2 (illusion of control), leads them to develop greater confidence in their ability to control the results.

Regarding Factor 2, the illusion of control refers to people’s tendency to believe they have more control over the outcomes of situations than they actually do. In the case of players with disabilities, there may be an increased propensity to experience this cognitive distortion; people with disabilities may feel the need to compensate for their limitation in other areas of their lives. Thus, through gambling, they may experience a sense of freedom and control that may be lacking in other areas, leading to a greater illusion of control. In turn, with the help of assistive technologies and adaptations, players with disabilities may have more control over their gaming environment (e.g., game control adaptations can allow them to play more easily, which reinforces the sense of control). Also, the social interaction and positive attention they receive online may reinforce the belief that they have control over the game.

Indeed, this may be in line with the results of a previous study [35]. The latter suggests that while students without disabilities may have common erroneous beliefs about gambling, such as the illusion of control and minimizing losses, students with disabilities may add additional challenges to these distortions due to a limited understanding of gambling and a greater reliance on luck due to their limitations, failing to appreciate the reality of the inherent risks involved in such participation.

## 5. Conclusions

From these results, this work reflects that students with disabilities represent an important risk group for the development of gambling addiction due to a combination of factors related to vulnerability, facilitated access, lack of support, and limited awareness of the risks involved. Likewise, improved accessibility through adaptive technologies and the lack of physical barriers may increase the participation of people with disabilities in gambling activities, thereby increasing the risk of developing pathological gambling.

Apart from that, for some students with disabilities, gambling can become a form of escape or entertainment, increasing the risk of developing a disordered relationship with gambling. Similarly, the lack of specific prevention and education programs for people with disabilities may leave this group without the necessary tools to recognize and address gambling problems. Finally, society and healthcare professionals may underestimate the risk of gambling addiction in people with disabilities, which can lead to a lack of detection and adequate treatment.

Despite the importance of this research, this study has several limitations. Firstly, the sample size is limited, which hinders the generalization of the results to the broader university population. Additionally, voluntary participation may introduce biases, as those students who choose to participate may have different characteristics or interests compared to those who do not, potentially affecting the validity of the results. Regarding the measurement instruments, although validated instruments like the GBQ were used, self-reported responses may be subject to social desirability biases or a lack of self-awareness. This could impact the accuracy of data on cognitive distortions and gambling participation.

Furthermore, the study may not have fully considered individual and contextual differences among students with and without disabilities, such as the type and severity of disability, social and family support, and access to resources, which can influence gambling behaviors and beliefs. Additionally, the lack of control or consideration of additional sociodemographic variables, such as socioeconomic status, marital status, and family history of addictions, may limit the ability to identify specific factors contributing to gambling addiction.

These limitations should be taken into account when interpreting the study results and underscore the need for future research with more robust designs, larger and more representative samples, and data collection methods that minimize biases. This would help obtain a more precise understanding of gambling addiction in university populations, both with and without disabilities.

In conclusion, it is evident that online games can have a significant impact on the cognition of students, both with and without disabilities. While these games offer learning and entertainment opportunities, they also pose risks of cognitive distortions, such as addiction and overstimulation. The findings can contribute to the development of more effective prevention and treatment strategies, informed by the specific characteristics and needs of students with disabilities, which is a valuable addition to the international literature on gambling addiction in disabled populations.

Research on online gambling addiction issues in university populations with disabilities in Spain is significant due to its inclusive and specific approach, its use of rigorous methods, and the relevance of its findings for intervention policies and programs. Moreover, it introduces several innovations in the literature, including data on an underrepresented population, a Spanish cultural perspective, and new insights into the interaction between cognitive distortions and gambling addiction in this demographic.

Additionally, this work can contribute to the study of online gambling addiction through theoretical implications that may help enrich and expand existing theories on addiction, stress and coping, self-efficacy, planned behavior, and emotional regulation, offering a more comprehensive and nuanced understanding of these phenomena.

Further studies in this line, both in Spain and other countries, are needed to propose a theoretical model that can understand and address behavioral addictions, providing useful perspectives for prevention, treatment, and intervention strategies tailored to the needs of populations with and without disabilities.

## Figures and Tables

**Table 1:** Exploratory factor analysis of gambling distortions.

		F1	F2
F2	3. My choices affect the game I’m betting on.	0.843	
F1	7. Gambling games are more than just luck.	0.823	
F1	6. When I’m gambling and I’m about to lose, or when I almost win, it reminds me that if I keep playing I’ll win.	0.808	
F1	4. I’m gambling and I’m losing, I should continue because I don’t want to miss out on a win.	0.805	
F1	15. It doesn’t matter where I get the money to gamble because I’ll win and pay it back.	0.801	
F2	20. I should keep the same bet even when it hasn’t come up lately because it’s destined or bound to win.	0.763	
F2	1. I think of gambling as a challenge.	0.732	
F1	5. I should keep track of previous winning bets to figure out how I should bet in the future.	0.687	
F1	14. Those who don’t gamble much don’t understand that success in gambling requires dedication and the willingness to invest some money.	0.687	
F2	12. There are certain things I do when I’m gambling (e.g., touching something a certain number of times, holding a lucky coin in my hand, crossing my fingers, etc.) that increase the chances of winning.	0.684	
F1	2. My knowledge and skill in the game contribute to the possibility of making money.	0.683	
F2	11. Even when I may be losing with my gambling strategy or technique, I should stick with it because I know I’ll win again.	0.675	
F1	9. I have a “lucky technique” that I use when I gamble.		0.895
F2	10. In the long run, I’ll make more money than I can lose gambling.		0.889
F1	8. My wins in gambling are proof that I have skill and knowledge about the game.		0.819
F2	17. If I keep playing over time, I’ll be rewarded and make money.		0.811
F1	18. I have more knowledge and skills related to gambling than most people who gamble.		0.738
F1	16. Gambling is the best way for me to experience excitement.		0.700
F1	13. If I lose money gambling, I should try to win it back.		0.663
F1	19. When I lose gambling, it’s not so bad if I don’t tell the people I care about.		0.632

Original GBQ model: F1 = luck/perseverance; F2 = illusion of control.

**Table 2:** Multinomial logistic regression analysis: parameter estimates for player typology.

							95% IC Exp (B)	
At-risk player	B	D. error	Wald	gl	Sig.	Exp (B)	L. inf	L. sup
[Dis. = 0]	−0.931	0.257	13.155	1	0.000	0.394	0.238	0.652
I should keep track of previous winning bets to figure out how I should bet in the future	−0.981	0.274	12.837	1	0.000	0.375	0.219	0.641
Those who don’t gamble much don’t understand that success in gambling requires dedication and the willingness to invest some money.	1.055	0.297	12.639	1	0.000	20.872	1.605	5.138
I have more knowledge and skills related to gambling than most people who gamble.	0.902	0.282	10.203	1	0.001	20.465	1.417	4.287
When I lose gambling, it’s not so bad if I don’t tell the people I care about.	−1.144	0.277	17.037	1	0.000	0.319	0.185	0.548
Problem player								
[Dis. = 0]	−1.541	0.417	13.648	1	0.000	0.214	0.095	0.485
My choices affect the game I’m betting on.	0.572	0.232	6.107	1	0.013	1.772	1.126	2.791
I should keep track of previous winning bets to figure out how I should bet in the future.	−1.120	0.549	4.165	1	0.041	0.326	0.111	0.957
When I’m gambling and I’m about to lose, or when I almost win, it reminds me that if I keep playing I’ll win.	−1.123	0.426	6.949	1	0.008	0.325	0.141	0.750
Those who don’t gamble much don’t understand that success in gambling requires dedication and the willingness to invest some money.	0.996	0.454	4.810	1	0.028	2.707	1.112	6.593
[Dis. = 0] * My knowledge and skill in the game contribute to the possibility of making money.	−2.309	0.877	6.925	1	0.009	0.099	0.018	0.555
[Dis. = 0] * Those who don’t gamble much don’t understand that success in gambling requires dedication and the willingness to invest some money.	−1.710	0.469	13.301	1	0.000	0.181	0.720	0.453

* The reference category is: no problems.

## Data Availability

Where no new data were created, or where data is unavailable due to privacy or ethical restrictions.
